# Structure‐based drug discovery facilitates future painkiller development

**DOI:** 10.1002/ctm2.1120

**Published:** 2022-11-22

**Authors:** Yuqing Li, Teng Li, Yang Du, Song Wu

**Affiliations:** ^1^ Department of Experiment & Research South China Hospital of Shenzhen University Shenzhen Guangdong China; ^2^ School of Medicine Kobilka Institute of Innovative Drug Discovery The Chinese University of Hong Kong Shenzhen Guangdong China

1

What drives people to seek for medical care? Pain may be the most common reason. This unpleasant sensory and emotional experience could be caused by a physiological response which is an essential early warning sign that usually elicits reflex withdrawal and thereby protects the organism from further injury, or pathological factors (e.g. chronic neuropathic pain). Pathological pain is an expression of the maladaptive operation of the nervous system, involving complex biopsychosocial interactions, causing tremendous disease burden.

The prevalence of chronic pain is estimated to range from 12% to 30% worldwide.^1^ Among various therapeutic approaches, pharmacotherapy is the most fundamental method to mitigate pain. Yet, currently available analgesics, including nonsteroidal anti‐inflammatory drugs (NSAIDs), amine reuptake inhibitors, antiepileptic drugs and opioids, are associated with deleterious side effects. Of note, opioids have been the mainstay of pain treatment since its advent. These drugs are recognised as the most efficient analgesics. However, they are inevitably coupled with severe limitations, for example, dependence, constipation and respiratory depression.^2^

Hence, the recent two centuries have witnessed the optimisation campaigns for more secure and efficient analgesics. Early attempts were made with conventional concepts, including modifying the existing validated analgesic drugs, changing the administration route and combination medication. As with the approaching of current biology, researchers turn to seek novel targets for analgesia drugs. However, inadequate understanding of mechanisms relevant for analgesia makes it difficult to find ideal therapeutic targets. Besides, experimental high‐throughput screening, as a part of the traditional drug development process, is often time‐consuming and laborious. Overall, the present assessment reveals the lack of real breakthroughs in analgesic drug development despite intense research efforts.^3^

The development of structural biology has led to a deeper understanding of the mechanisms of pain and analgesics. It is now clear that morphine targets the μ‐opioid receptor (μOR), by activating G protein Gi which mediates analgesia; while inevitably in couple with β‐arrestin signalling in the downstream of μOR, which confers lethal side effects. To disentangle these signals, several G‐protein‐biased agonists have been developed in the 21st century, including the compound PZM21.^4^ By computational docking and subsequent modification, Manglik and his colleagues developed this molecule structurally distinct from previous opioid ligands, with a strong analgesic effect (EC50 = 4.6 nM) while devoid of many of the side effects of current opioids.

Although the strategy to develop biased agonists of μOR casts light on analgesic drug development, novel candidates that are capable of disassociating analgesia and repression signals are somewhat fortuitous. Moreover, the remaining activity to recruit β‐arrestin leaves a gap between PZM21 and the ideal pain killer. Towards this aim, recently Fink and her colleagues have achieved a milestone success in developing nonopioid analgesics.^5^ Ligands of a nonopioid receptor, α_2A_‐adrenergic receptor (α_2A_AR), have been clinically available, such as dexmedetomidine. However, their strong sedative effects and limited administration routes hinder future therapeutics targeting this receptor.^6^ Through structure‐based docking, Fink et al. computed more than 301 million virtual molecules against the α_2B_‐adrenergic receptor (α_2B_AR), which harbours a more conserved binding site than α_2A_AR. Among the most promising compounds, several agonists selectively activate Gi, G_o_, and G_z_ G protein subtypes, chemically unrelated to known α_2A_AR drugs (Figure 1). Following a series of experiments and modifications, some of these novel α_2A_AR agonists, especially ‘9087 and PS75, successfully separate the analgesic effect from sedation without recruitment of β‐arrestin or receptor internalisation. In summary, this work springs some lead agonists for future development of nonopioid pain killers.

**FIGURE 1:**
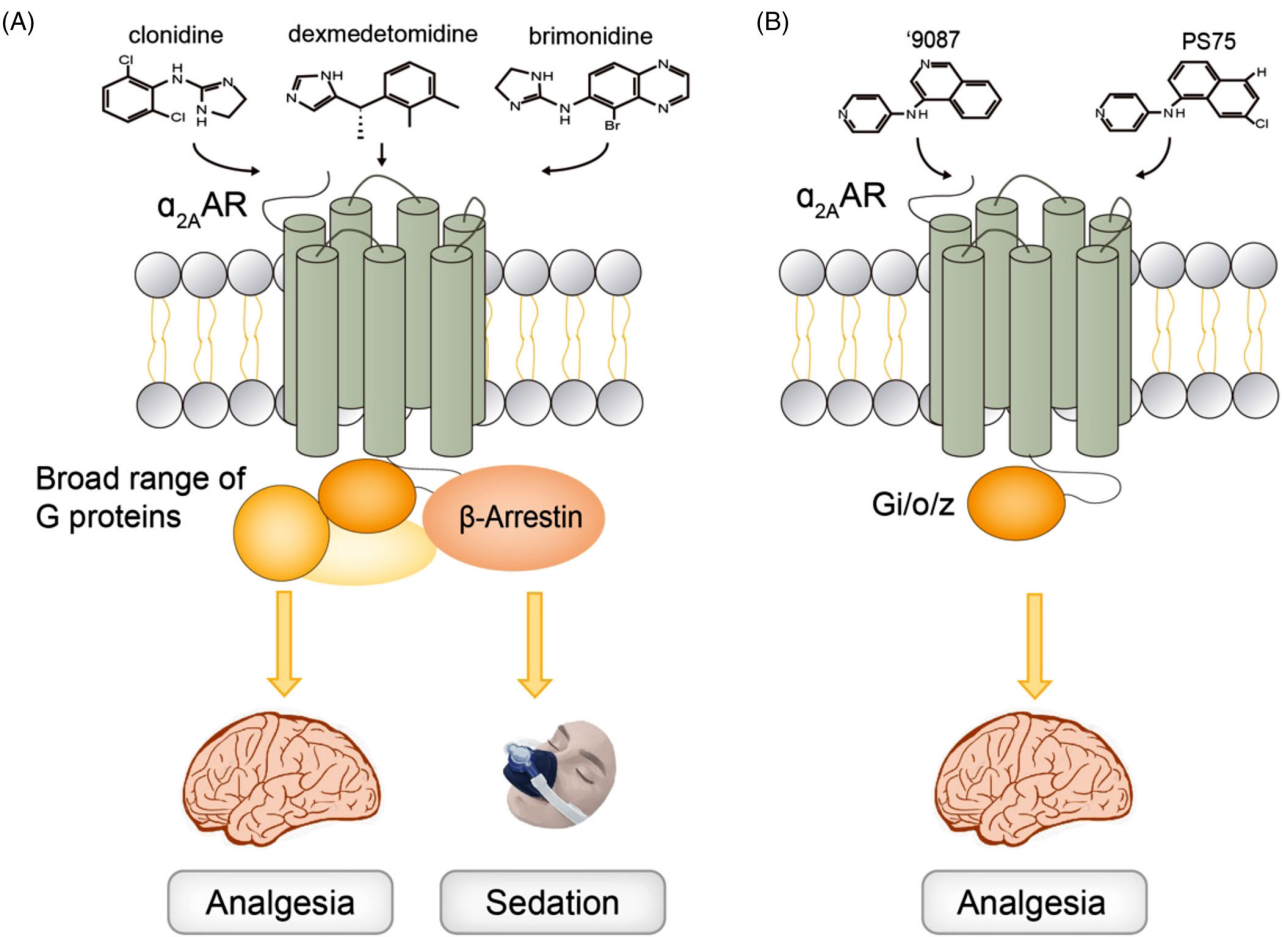
Schematic diagram of conventional α2AAR drugs and newly discovered agonists. (A) The known therapeutics targeting the α_2A_AR, like clonidine, dexmedetomidine and brimonidine, broadly activate G proteins and recruit β‐arrestins, inducing both analgesia and sedation. (B) The docking‐derived agonists discovered by Fink et al.,^5^ like ‘9087 and PS75, preferentially activate Gi/o/z G protein subtypes without engagement of β‐arrestins, separating analgesic properties from sedation

Some principles of drug development strategies could be distilled from this most updated study. Taking advantage of virtual libraries allows us to identify novel and promising scaffolds from millions to billions of tangible compounds, most of which have not been previously synthesised. Improvement of in vivo activity could be further optimised based on these scaffolds through extensive structure‐activity optimisation. Second, although new chemotypes are not logically associated with new signalling pharmacology, this concept does increase the chances to get agonists with more selective activities compared to existed drugs. Much crucially, the selection of the G protein receptor with conserved orthosteric sites contributes to the achievement of efficacious analgesics that disentangle from sedative effect. Such orchestration is also expected to bring insights into developing other drugs with more robust therapeutic and minor side effects.

Several caveats must be emphasised for these structure‐based drug campaigns. While the novel α_2A_AR agonists do disentangle from known lethal side effects, how this new pharmacology is implemented remains obscure. The minor deviation between the engagement of G‐proteins and β‐arrestins and the orthosteric site, suggests that the effects of other unknown pathways should not be excluded. Thus, further experimental work on the molecular mechanism as well as a functional study on multiple preclinical models should be launched. Nevertheless, the work by Fink et al. provides a paradigm for structure‐based drug discovery targeting pharmacological important GPCRs.

## CONFLICT OF INTEREST

The authors declare no conflict of interest.
